# Enteroenteric intussusception in an adult caused by an ileal angiomyolipoma

**DOI:** 10.1590/0100-3984.2014.0143

**Published:** 2015

**Authors:** Rodolfo Mendes Queiroz, Luana Almeida Botter, Michela Prestes Gomes, Rafael Gouvêa Gomes e Oliveira

**Affiliations:** 1 Documenta – Hospital São Francisco, Ribeirão Preto, SP, Brazil.; 2 Faculdade de Medicina de Ribeirão Preto da Universidade de São Paulo (FMRP-USP), Ribeirão Preto, SP, Brazil.

*Dear Editor,*

A white, 32-year-old man was admitted to the emergency department with severe pain principally in the right inferior quadrant of the abdomen, abdominal distension and vomiting for one day.

**Figure 1:**
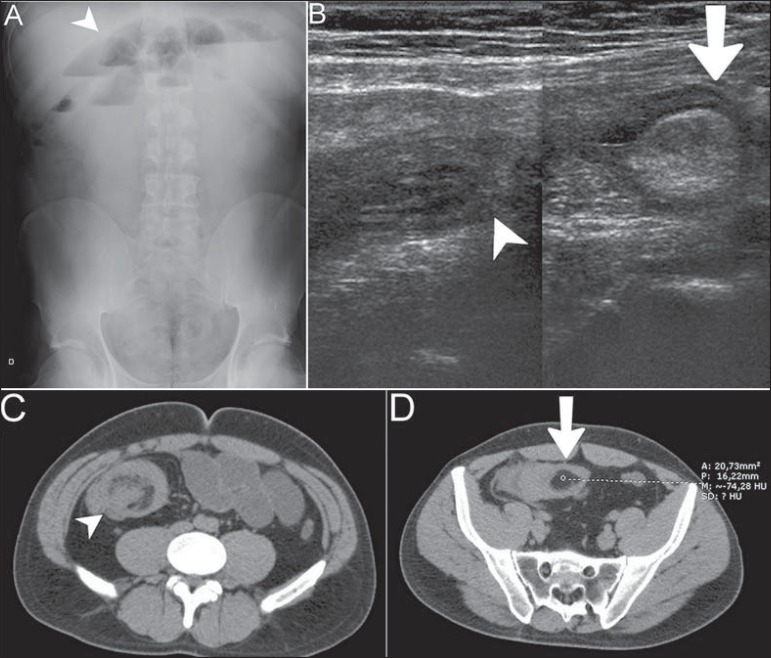
**A:** Radiographic image demonstrating small bowel loops distension with fluid levels (arrowhead). **B:** Ultrasonography images composition showing invagination of intestinal wall (arrowhead) adjacent to an echogenic intraluminal nodule (arrow). **C,D:** Contrastenhanced computed tomography, precontrast phase showing target sign (arrowhead) representing an intussusception adjacent to intraluminal nodule with fat density (arrow).

Abdominal radiography, ultrasonography and computed tomography demonstrated small bowel loops distension (Figure 1A) and signs of ileo-ileal invagination associated with intraluminal nodule with fat content compatible with “intussusception head” (Figures 1B, 1C and 1D). Option was made for surgical treatment.

Anatomopathological study in association with immunohistochemical analysis diagnosed angiomyolipoma (AML) as follows:

*Macroscopy:* Bowel loop containing a non-encapsulated delimited, submucosal, polypoid yellowish lesion measuring 3.0 × 2.5 × 2.3 cm, with no sign of malignancy.

*Microscopy:* Masson’s trichrome staining diagnosed AML compromising the entire intestinal wall, from the serosa to the mucosa.

*Immunohistochemical analysis:* Desmin, HHF 35, CD31, CD34, protein S100, smooth muscle actin 1 to 4 = positive.

Intussusception is the invagination of a proximal intestinal segment with its mesenteric fold with the corresponding vascu- larization into the lumen of the distal intestinal portion, which may lead to obstruction, inflammatory process and segmental ischemia ^(1,2)^.

In adult individuals, this condition corresponds to about 5% of the general cases, out of which only 1% of cases cause obstruction ^(1)^. In this age group, it is estimated that in 90% of cases one finds organic intraluminal causes called “intussusception heads” (for example, benign neoplasms such as lipoma, or malignant; adenomatous polyps or other polyp types; hamartomas) or extraluminal causes (for example, adhesions, Meckel’s diverticulum)^( 1,3)^.

Intussusception heads in the small bowel are most frequently associated with benign lesions, while in the colon they are most associated with either primary or secondary malignant neoplasias ^(2)^. The treatment is generally surgical for organic causes, complications such as obstruction and intestinal ischemia^(2,3)^.

The clinical signs of intussusception are related to the occurrence of subocclusion, obstruction and enterorrhagia^(3,4)^.

Intussusceptions are classified according the involved intestinal segment, as follows: enteroenteric, colo-colic, ileocolic and ileocecal intussusception^(1,3)^.

Typical radiological findings include: “target sign” and “pseudokidney sign”^(3)^. The diagnostic accuracy of ultrasonography approximates to 98%^(2)^, but the method is operator-dependent^(5)^. Computed tomography presents accuracy of 58% to 100%^(5)^.

AMLs are benign mesenchymal tumors containing adipose, smooth muscle, epithelial and vascular cells^(4,6–8)^. These tumors and other lesions such as lymphangioleiomyomatosis and clear cell lung tumors were brought together under the classification of PEComas (perivascular epithelioid cell tumors)^(4)^.

The prevalence of renal angiomyolipomas ranges from 0.3% to 3%. According to the literature this tumor is sporadic in about 80% of cases, and the remaining cases are associated with lymphangioleiomyomatosis and mainly tuberous sclerosis^(6,7)^.

Extrarenal AMLs are extremely rare, and the liver is the most reported site (some other locations include the heart, lungs, retroperitoneum, mediastinum, spinal cord, mucocutaneous, parotid glands, reproductive organs regardless of sex), and its occurrence in the gastrointestinal tract is rarely described^(4,6–8)^ (about 50 cases)^(6,7)^.

When located in the gastrointestinal tract their radiological diagnosis is hardly achieved because of their rarity and adipose nature, similarly to lipomas which are much more frequently found^(4,7,8)^.
